# Tratamento endovascular de aneurisma de aorta abdominal com fístula aorto-cava utilizando oclusor vascular concomitante a endoprótese bifurcada: relato de caso

**DOI:** 10.1590/1677-5449.007916

**Published:** 2017

**Authors:** Bruno Lorenção de Almeida, Fabio Henrique Rossi, Thiago Osawa Rodrigues, Leandro Berutto Ahouagi, Sthefanie Fauve Andrade Cavalcante, Camila Bauman Beteli, Carlos Augusto Cardoso Pedra, Antônio Massamitsu Kambara

**Affiliations:** 1 Instituto Dante Pazzanese de Cardiologia – IDPC, São Paulo, SP, Brasil.

**Keywords:** aneurisma aórtico, fístula aorto-cava, procedimentos endovasculares

## Abstract

As fístulas aorto-cava são entidades raras e de etiologia variada, estando frequentemente associadas a significativa morbimortalidade. Acredita-se que o aumento da tensão da parede nos grandes aneurismas resulte em reação inflamatória e aderência à veia adjacente, culminando na erosão das camadas aderidas e na formação da fístula. O tratamento cirúrgico convencional tem altas taxas de mortalidade. Embolia pulmonar paradoxal e o vazamento são complicações temidas do tratamento endovascular. O uso de oclusor vascular associado a endoprótese bifurcada é boa opção no tratamento do aneurisma de aorta abdominal com fístula aorto-cava.

## INTRODUÇÃO

As fístulas aorto-cava são entidades raras e de etiologia variada associadas a significativa morbimortalidade. A imensa maioria resulta da erosão ou ruptura de aneurismas da aorta abdominal para a veia cava inferior. O objetivo deste artigo é apresentar o tratamento endovascular de um caso de fístula aorto-cava em paciente com aneurisma de aorta abdominal utilizando oclusor vascular associado a uma endoprótese bifurcada.

## DESCRIÇÃO DO CASO

Paciente masculino, 71 anos, tabagista e ex-etilista, com diagnóstico de aneurisma de aorta abdominal infrarrenal há 15 anos, sem acompanhamento regular, foi encaminhado ao Serviço de Cirurgia Endovascular de nossa instituição para avaliação e possível tratamento. Relatou aparecimento de massa abdominal pulsátil, associada a dor abdominal difusa, intermitente e de longa data. Referia ainda edema de membros inferiores há 8 meses, adinamia e emagrecimento de 20 kg nos últimos 6 meses. Ao exame, apresentava massa pulsátil em mesogástrio, com abdome difusamente doloroso à palpação e com presença de frêmito em flanco esquerdo.

**Figura 1:**
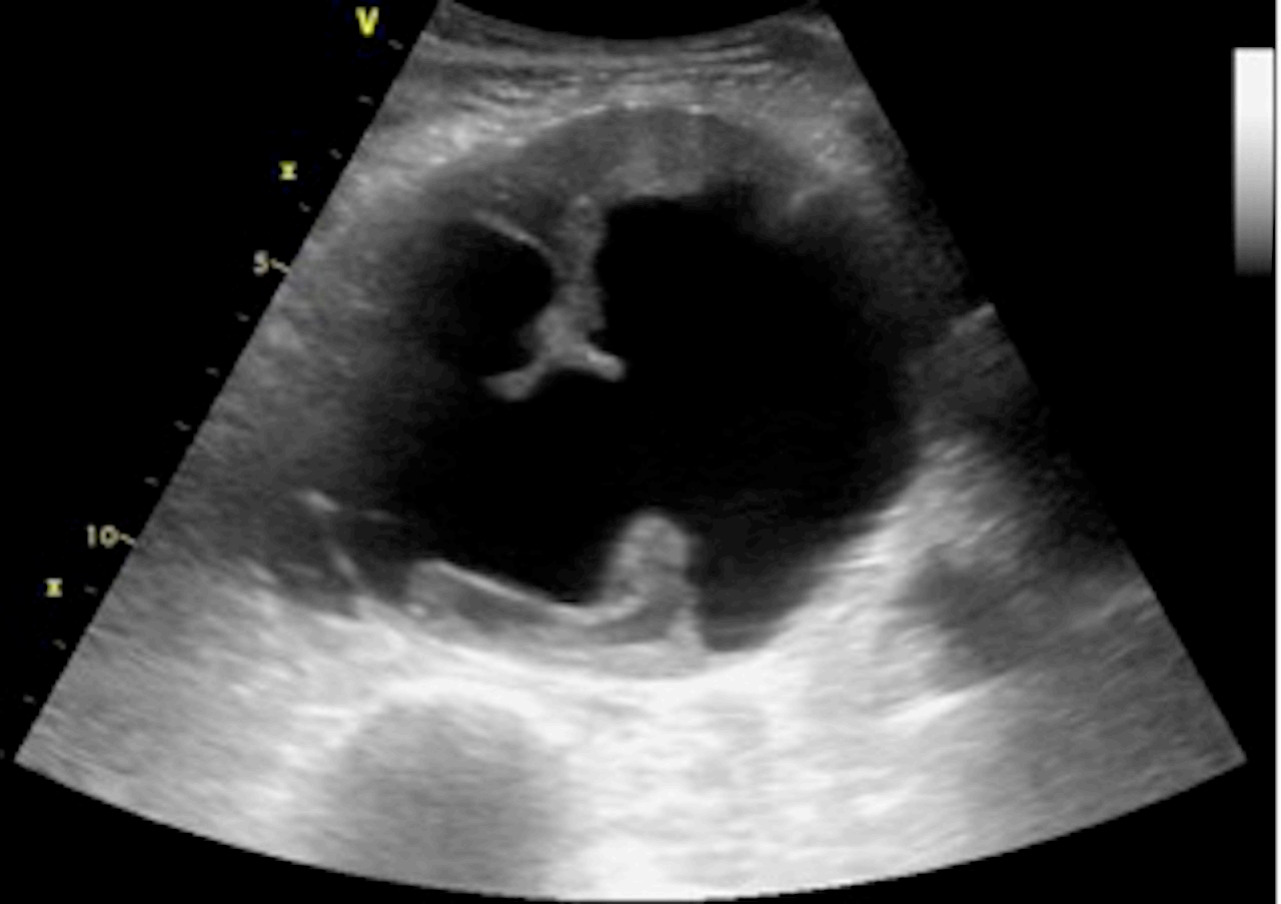
Aneurisma abdominal com imagem de trombos murais ao ultrassom em modo B.

**Figura 2:**
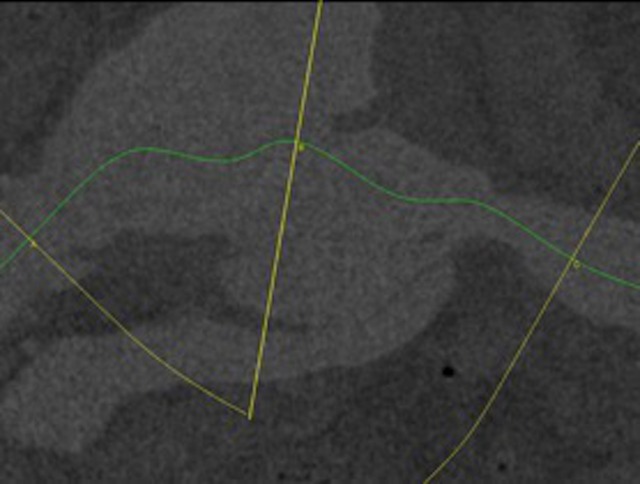
Aneurisma de aorta abdominal com fístula aorto-cava à angiotomografia.

Ultrassonografia abdominal com Doppler colorido demonstrou aneurisma de aorta abdominal com 9,7 cm de diâmetro com presença de trombo mural e trombos móveis em sua luz (Figura 1). Na parede posterolateral direita, observou-se fluxo de alta velocidade, sugerindo fístula arteriovenosa com 5 mm de diâmetro, comunicando o aneurisma com a veia cava inferior. Angiotomografia de aorta evidenciou dilatação aneurismática, fusiforme da aorta abdominal infrarrenal estendendo-se até a bifurcação das artérias ilíacas comuns e medindo 9,2 cm, além da presença de comunicação entre aorta abdominal e veia cava inferior em sua parede posterolateral direita medindo cerca de 8 mm de diâmetro e localizada 2 cm acima da bifurcação ilíaca (Figura 2). Notou-se ainda aumento de câmaras cardíacas direitas e derrame pleural com atelectasia de lobos pulmonares inferiores bilateralmente. Apesar da presença de dilatação das câmaras cardíacas na tomografia, o ecocardiograma evidenciou aumento atrial discreto e função cardíaca preservada.

Optamos pelo tratamento endovascular com endoprótese bifurcada e oclusor vascular, após consentimento do paciente, dada a menor morbimortalidade e condições anatômicas e clínicas favoráveis à realização do procedimento. O tratamento se iniciou pela dissecção bilateral das artérias femorais comuns e pelo posicionamento de introdutor valvulado 6F bilateralmente, sob anestesia geral e monitorização cardiopulmonar. Foi realizada punção de veias femorais comuns e foram posicionados introdutores valvulados 5F bilateralmente. Foi posicionado cateter Pigtail centimetrado na aorta abdominal pelo acesso arterial direito e fio guia Lunderquist 0,035, 300cm pelo acesso arterial esquerdo, para retificação da anatomia aórtica.

**Figura 3:**
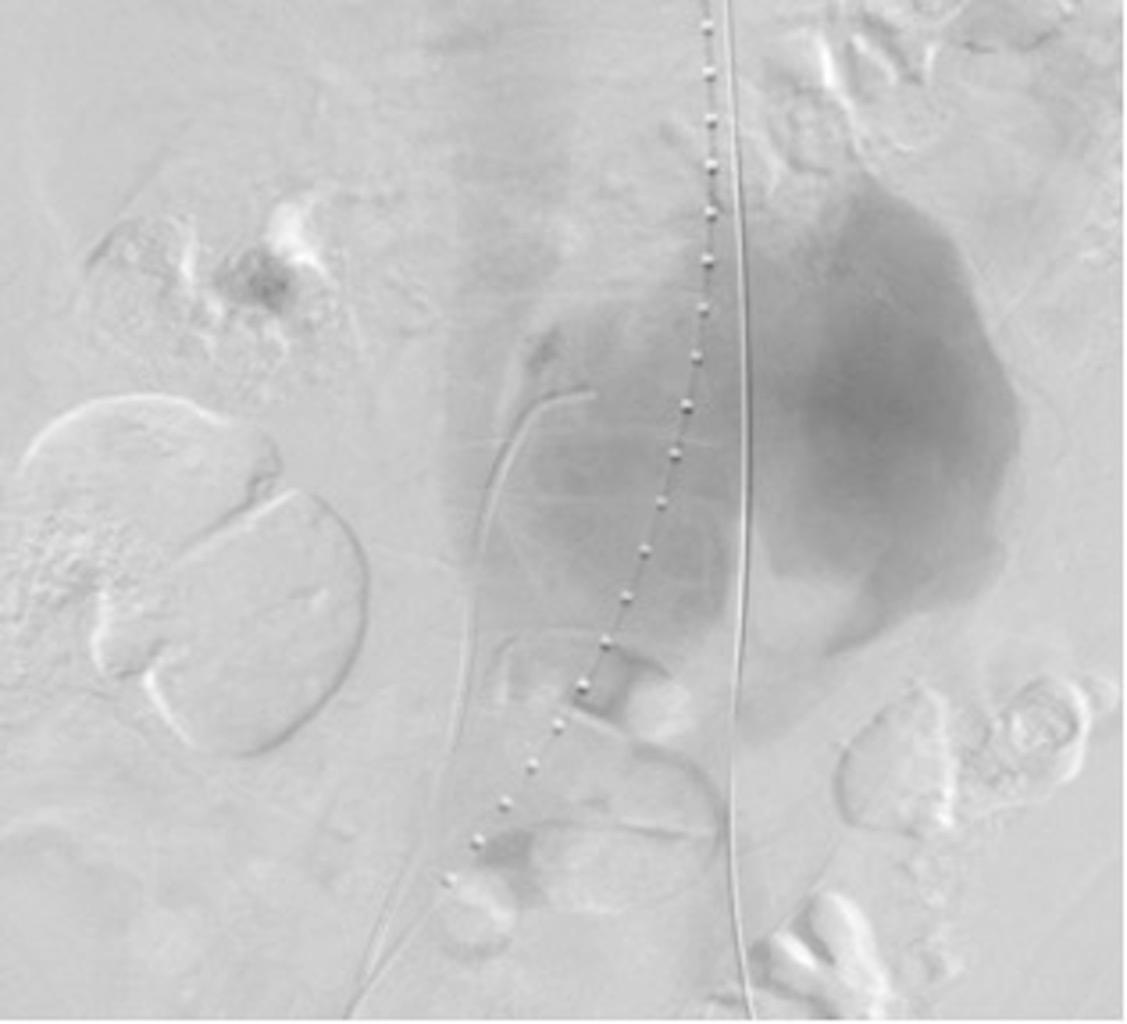
Trajeto fistuloso cateterizado com cateter JR 5F pelo acesso venoso direito.

**Figura 4:**
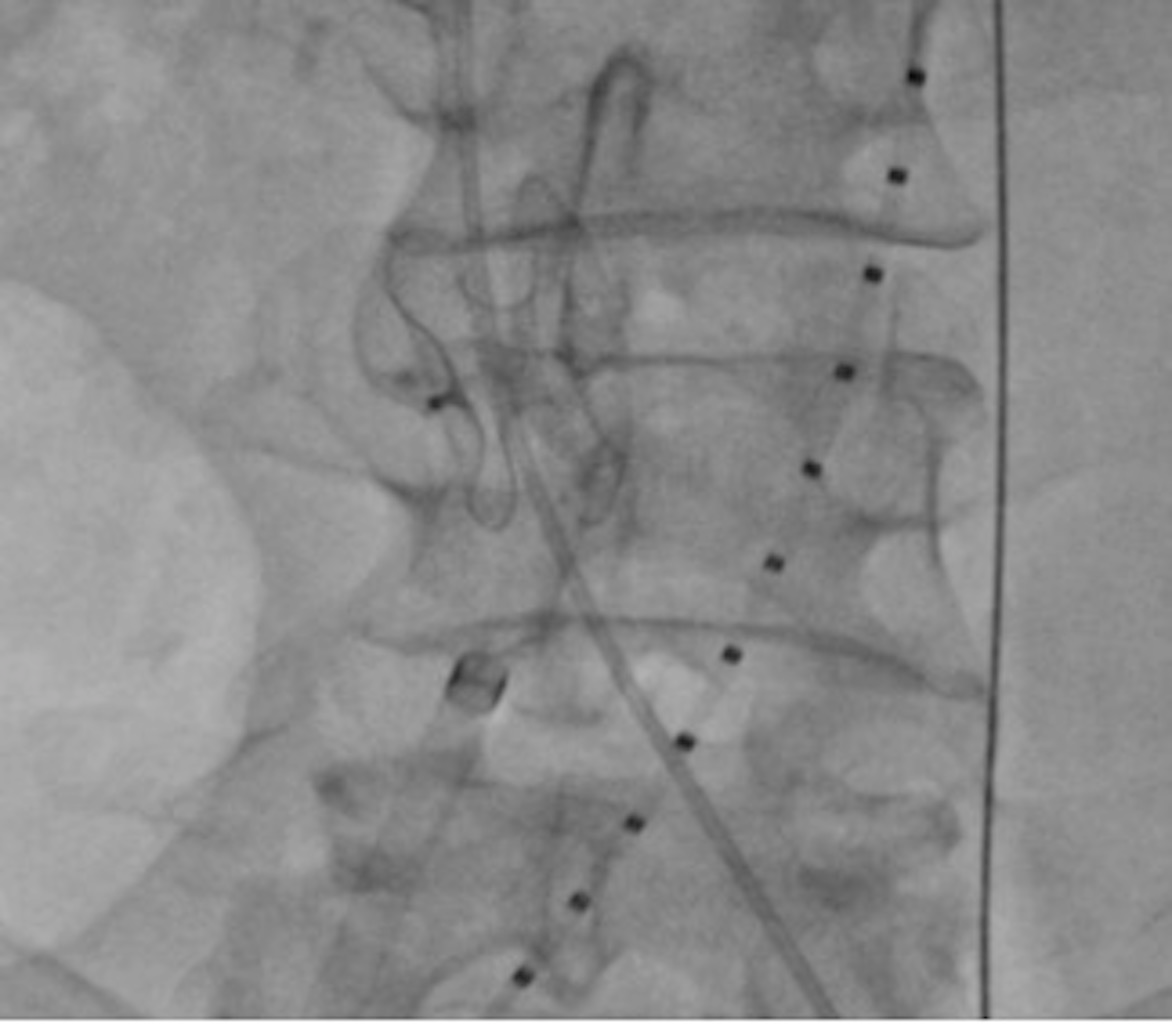
Oclusor Figulla II em posição após liberação.

Realizou-se flebografia inicial, que evidenciou circulação colateral exuberante, proveniente das veias ilíacas internas, compressão extrínseca da veia cava inferior no seu segmento distal – pelo aneurisma adjacente – e imagem compatível com fístula artério-venosa nessa topografia (Figura 3). Foi realizada cateterização do trajeto fistuloso pelo acesso venoso direito com cateter JR diagnóstico 5F e guia hidrofílico 0,035, posteriormente trocado por guia Amplatz extra stiff 0,035, 260cm. Uma bainha Flexor Check-Flo 12F 45cm (Cook) foi posicionada através do orifício da fístula, pelo acesso venoso direito. Nesse momento, foi posicionado e liberado oclusor vascular Figulla flex II 21mm (Occlutech) com dois discos concêntricos, obtendo sucesso na oclusão do trajeto fistuloso entre a aorta e a veia cava inferior (Figura 4). O tamanho do oclusor se baseou no tamanho do orifício fistuloso, mensurado nas angiotomografia e angiografia iniciais, sendo sobredimensionado para garantir uma boa aposição à parede degenerada da aorta, evitando migração.

**Figura 5:**
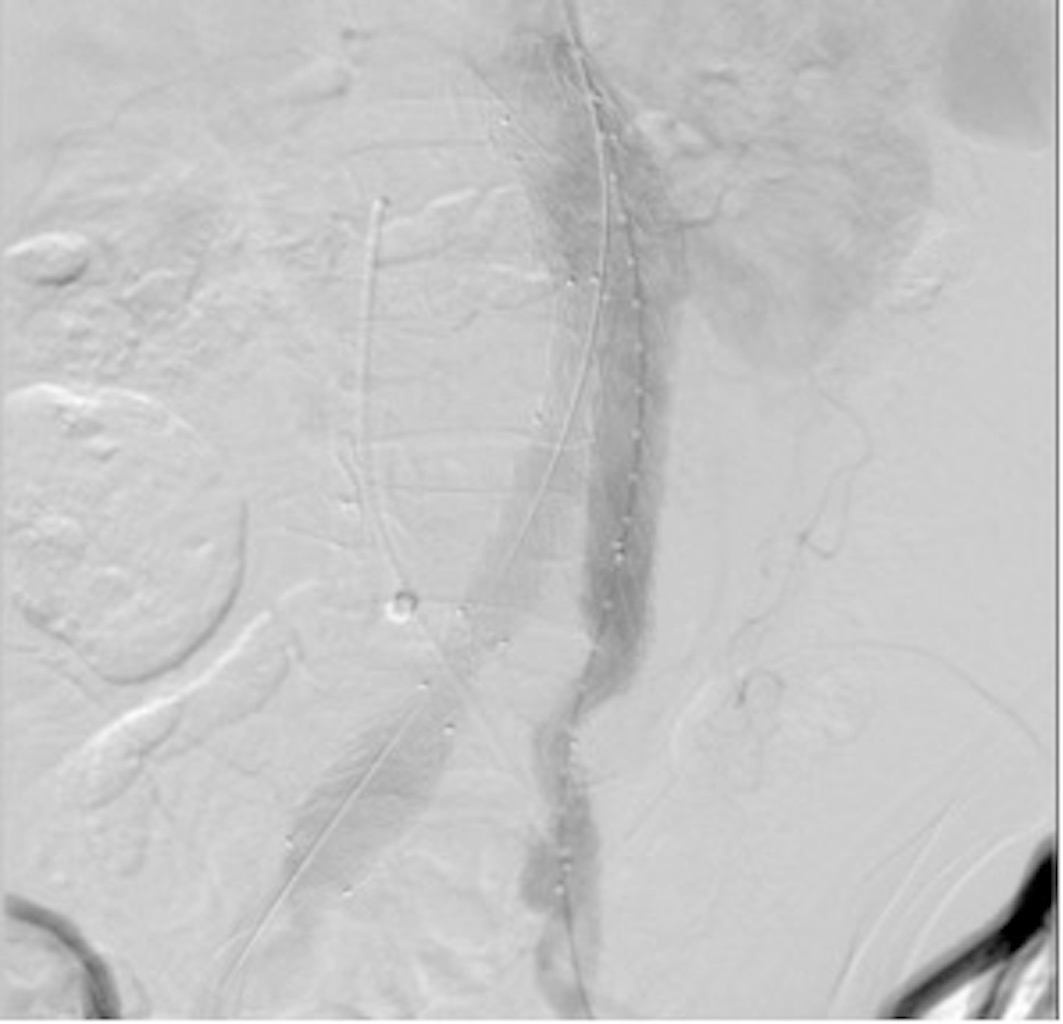
Angiografia final sem evidência de vazamentos.

Realizou-se então o tratamento do aneurisma de aorta abdominal infrarrenal, com corpo principal de endoprótese Endurant (Medtronic) 36×20×166 mm pelo acesso arterial esquerdo, com extensões 16×16×124 mm e 16×24×82 mm contralateral e 16×20×93 mm ipsilateral. Angiografia final evidenciou sucesso no tratamento do aneurisma, perviedade de artérias renais e ausência de vazamentos, mesmo após injeção concomitante pelos acessos arterial e venoso (Figura 5).

**Figura 6:**
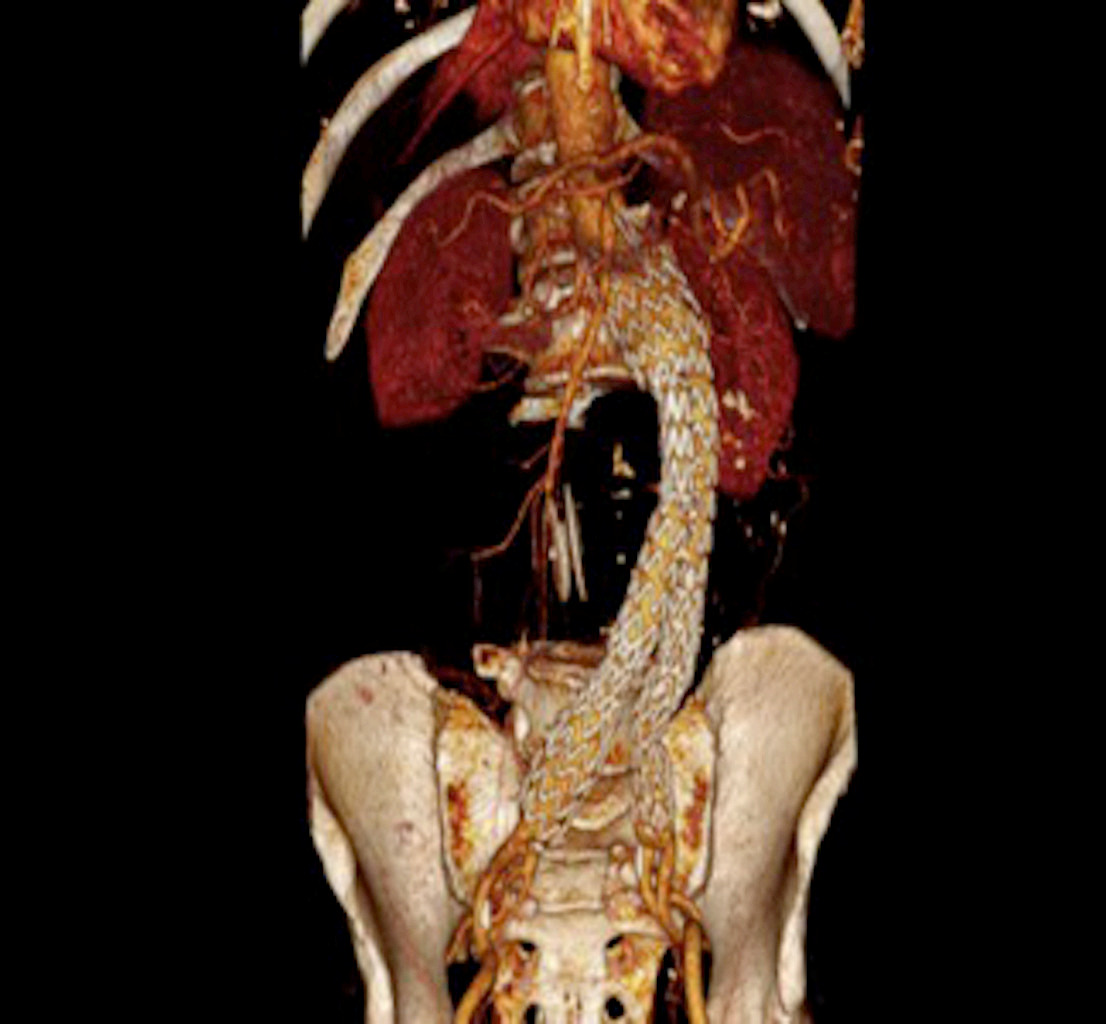
Angiotomografia com reconstrução 3D do acompanhamento de 30 dias.

O paciente apresentou recuperação adequada no pós-operatório, recebendo alta no quinto dia, em boas condições clínicas e com edema de membros inferiores em regressão. Angiotomografia de controle aos 30 dias evidenciou endoprótese pérvia, sem sinais de vazamentos. Observou-se também veia cava inferior pérvia e oclusor bem posicionado, sem evidência de trombose secundária (Figura 6). Já se passa 1 ano do tratamento e infelizmente o paciente se nega a realizar qualquer retorno ambulatorial ou a realizar exames de imagem. Em contato telefônico, informa permanecer sem novas queixas ou sintomas relacionados.

## DISCUSSÃO

A fístula aorto-cava é uma rara complicação dos aneurismas de aorta abdominal infrarrenal, podendo chegar a 4% dos casos de aneurisma roto^1^. O primeiro caso relatado foi publicado por Syme em 1831, sendo que, em 1955, Cooley relatou seu tratamento cirúrgico com sucesso^2^.

Acredita-se que o aumento da tensão da parede dos aneurismas resulte em reação inflamatória e aderência à veia adjacente – geralmente a veia cava inferior – culminando na erosão das paredes e formação da fístula^3^. Sua apresentação clássica consiste em massa abdominal pulsátil associada a frêmito e “sopro em maquinaria”, insuficiência cardíaca direita e sinais de hipertensão venosa. Raramente pode ocorrer embolia pulmonar paradoxal (EPP), ocasionada pela passagem de trombos do aneurisma para a circulação venosa^4^. Outros sinais e sintomas incluem estase jugular, dispneia, derrame pleural, hepatomegalia, ascite, hematúria^5^.

Devido à sua gravidade, as fístulas aorto-cava devem ser abordadas tão logo seja feito o diagnóstico. Com o tratamento cirúrgico convencional, a mortalidade perioperatória gira em torno de 16 a 66%^6^. Isso se deve ao fato de se tratar de pacientes normalmente em idade avançada, com múltiplas comorbidades, complicadas pelas alterações hemodinâmicas sistêmicas que uma fístula de alto débito provoca^7^. Avaliação pré-anestésica minuciosa da função cardiopulmonar, bem como administração criteriosa de fluidos e controle pressórico são essenciais para aumentar as chances de sucesso e diminuir as complicações, principalmente no momento do fechamento da fístula, quando pode ocorrer descompensação cardíaca aguda^8^.

As técnicas endovasculares são atrativas frente ao tratamento cirúrgico convencional. Revisão de literatura publicada por Antoniou et al. em 2009 mostra sucesso técnico de 96% no tratamento por via endovascular, sem relato de mortalidade perioperatória em 30 dias^9^. Porém, existem algumas preocupações teóricas relacionadas ao tratamento endovascular. Primeiramente, a manipulação da luz do aneurisma poderia provocar o deslocamento de trombos e uma EPP. Além disso, o tratamento do aneurisma sem a oclusão da fístula poderia predispor ao vazamento, pela persistência do canal fistuloso^10^.

A EPP é um evento raro porém com alta morbimortalidade^11^. Por ser de diagnóstico difícil e se confundir com os sintomas de insuficiência cardíaca do paciente, pode ser subestimada^12^. Alguns relatos descreveram a utilização de filtro de veia cava temporário para evitar a embolia paradoxal durante a manipulação da luz do aneurisma para o posicionamento da endoprótese^13^^,^ 14. Essa prática, entretanto, não se repetiu com frequência na literatura. Outros relatos apenas fizeram o tratamento convencional do aneurisma com endoprótese, sem a utilização de filtros, obtendo sucesso no fechamento da fístula aorto-cava sem relato de embolia paradoxal^15^^-^ 17. Devido ao tamanho do aneurisma rechaçando a parede da veia cava (o que poderia dificultar o implante de filtro temporário e sua retirada) e dada a disponibilidade do oclusor, optamos por não utilizar o filtro. Assim, ao ocluir o trajeto fistuloso previamente à introdução da endoprótese, manipulamos minimamente a luz aneurismática, evitando o deslocamento de trombos e consequentemente a EPP.

O vazamento tipo II – aquele proveniente de fluxo retrógrado de ramos do aneurisma, tipicamente de artéria lombar ou mesentérica inferior – é a complicação mais encontrada no tratamento endovascular das fístulas aorto-cava, presente em até 22% dos casos^9^. Entretanto, relatos da literatura mostram se tratar de evento normalmente autolimitado^18^. Esse tipo de vazamento parece estar sujeito a uma dinâmica diferente, na qual a baixa pressão do território venoso favorece uma via de saída para o fluxo retrógrado dos ramos aórticos, diminuindo a tensão na parede aórtica e favorecendo sua resolução espontânea^19^. Entretanto, alguns autores sugerem que, mesmo após liberação da endoprótese, pode haver fluxo exacerbado de sangue para dentro do saco aneurismático através da fístula, o que levaria a um segundo procedimento para sua correção. Pensando nisso, ElKassaby et al. e Silveira et al. propuseram o tratamento concomitante do aneurisma e da fístula aorto-cava, com a utilização de endoprótese do lado arterial e venoso, o que se mostrou factível, podendo ser mais efetivo que o tratamento endovascular aórtico exclusivo^20^^,^ 21. Levando em conta o tamanho do aneurisma e da fístula, o risco de endoleak nos pareceu muito alto e optamos pelo tratamento de ambos em um só tempo. Caso a fístula não fosse ocluída e surgisse um vazamento durante o acompanhamento, seu tratamento iria requerer outra estratégia, provavelmente com gasto de mais material de alto custo, além do risco de uma nova abordagem invasiva para o paciente. Havendo o material disponível para tratamento em um só tempo cirúrgico, julgamos mais seguro fazê-lo.

Apesar de seu uso *off-label* neste caso, o oclusor se adaptou bem às paredes arterial e venosa, cumprindo seu papel sem apresentar grandes dificuldades técnicas em seu posicionamento e liberação, uma vez que se tenha cateterizado o trajeto fistuloso. Oclusores vasculares já vêm sendo utilizados em pacientes submetidos a implante percutâneo de válvula aórtica que tenham o eixo iliofemoral afilado, nos quais a confecção de um trajeto fistuloso entre a veia cava e a aorta é opção para a ascensão de dispositivos de grande diâmetro^22^^,^ 23. Godart et al.^24^ e LaBarbera et al.^25^ utilizaram um oclusor Amplatzer para tratamento da fístula aorto-cava. Entretanto, em seus relatos os autores utilizaram o dispositivo oclusor como procedimento de resgate em pacientes previamente tratados com endoprótese ou cirurgia convencional para correção do aneurisma abdominal e que apresentaram fluxo persistente pelo orifício fistuloso no acompanhamento.

O uso de oclusor vascular associado a endoprótese bifurcada para tratamento de caso de aneurisma de aorta abdominal infrarrenal com fístula aorto-cava teve sucesso com resultado satisfatório imediato. Outros estudos são necessários para avaliar o uso rotineiro de oclusores vasculares no tratamento de fístulas aorto-cava e seu seguimento em longo prazo. Com a evolução dos materiais endovasculares, novos oclusores ou endopróteses para uso venoso exclusivo podem se tornar a primeira escolha no tratamento de fístulas aorto-cava.
