# Supplementary material for: Tratamento endovascular de aneurisma de aorta abdominal com fístula aorto-cava utilizando oclusor vascular concomitante a endoprótese bifurcada: relato de caso
Source: J Vasc Bras. 2017 Apr-Jun;16(2):168–73. [Article in Portuguese] doi: 10.1590/1677-5449.007916 (PMC5915865; doi:10.1590/1677-5449.007916)
Supplement: Translation

# Translation: Endovascular treatment of an abdominal aortic aneurysm with aortocaval fistula using a vascular occluder and a bifurcated endograft in a single intervention: case report

**Authors:** Bruno Lorenção de Almeida, Fabio Henrique Rossi, Thiago Osawa Rodrigues, Leandro Berutto Ahouagi, Sthefanie Fauve Andrade Cavalcante, Camila Bauman Beteli, Carlos Augusto Cardoso Pedra, Antônio Massamitsu Kambara

**Affiliations:** 1 Instituto Dante Pazzanese de Cardiologia – IDPC, São Paulo, SP, Brazil.

**Keywords:** aneurysm aortic, aortocaval fistula, endovascular procedures

## Abstract

Aortocaval fistulae are rare entities with a variety of etiologies and are very often associated with significant morbidity and mortality. It is believed that increased tension in the walls of large aneurysms can cause an inflammatory reaction resulting in adhesion to the adjacent vein and culminating in erosion of the adherent layers and fistula formation. Conventional surgical treatment has high mortality rates. Paradoxical pulmonary embolism and endoleaks are the most concerning complications linked with endovascular treatment. Using a vascular occluder in combination with a bifurcated endograft is a good option for the treatment of an abdominal aortic aneurysm with aortocaval fistula.

## INTRODUCTION

Aortocaval fistulae are rare entities with a variety of etiologies and are associated with significant morbidity and mortality. The great majority are the result of erosion or rupture of an abdominal aorta aneurysm into the inferior vena cava. The objective of this article is to describe a case of aortocaval fistula in a patient with an abdominal aortic aneurysm that was managed with endovascular treatment using a vascular occluder combined with placement of a bifurcated endograft.

## CASE DESCRIPTION

A 71-year-old male patient who was a smoker with a history of drinking and a preexisting infrarenal abdominal aortic aneurysm diagnosed 15 years previously, but not monitored regularly, was referred to the Endovascular Surgery Service at our institution for evaluation and possibly for treatment. He complained of an abdominal pulsating mass, associated with diffuse abdominal pains that were intermittent and had had onset a long time previously. He also reported edema of the lower limbs over the previous 8 months, asthenia, and weight loss of 20 kg over the preceding 6 months. Examination revealed a pulsating mass in the mesogastrium, with abdomen diffusely painful on palpation and a perceptible thrill in the left flank.

Abdominal color Doppler ultrasonography indicated an abdominal aortic aneurysm with a diameter of 9.7 cm, with mural thrombus and mobile thrombi in the lumen (Figure 1). High velocity flow was observed at the right posterolateral wall, suggestive of an arteriovenous fistula with a diameter of 5 mm, communicating between the aneurysm and the inferior vena cava. Angiotomography of the aorta showed aneurysmal dilatation, fusiform at the infrarenal abdominal aorta, extending to the bifurcation of the common iliac arteries and measuring 9.2 cm, in addition to communication between the abdominal aorta and the inferior vena cava at the right posterolateral wall, measuring around 8 mm in diameter and located 2 cm above the iliac bifurcation (Figure 2). The right cardiac chambers were also enlarged and there was pleural effusion with atelectasis of the lower pulmonary lobes, bilaterally. Despite the presence of cardiac chamber dilatation seen on tomography, the echocardiogram showed discrete atrial enlargement and preserved cardiac function.

**Figure 1:** Abdominal aneurysm with image showing mural thrombi on B mode ultrasound.

**Figure 2:** Abdominal aortic aneurysm with aortocaval fistula shown by angiotomography.

We decided, with the patient’s consent, to undertake endovascular treatment with a bifurcated endograft and vascular occluder, in view of the lower morbidity and mortality and the favorable anatomic and clinical conditions for the procedure. The first step was bilateral dissection of the common femoral arteries and placement of 6Fr valved introducers bilaterally, under general anesthesia and with cardiopulmonary monitoring. The common femoral veins were punctured and 5Fr valved introducers were placed bilaterally. A graduated Pigtail catheter was introduced into the abdominal aorta via the right arterial access and a 300cm 035 Lunderquist guide wire was introduced via the left arterial access, to straighten the aortic anatomy.

Initial phlebography revealed strong collateral circulation, originating from the internal iliac veins, extrinsic compression of the distal segment of the inferior vena cava – by the adjacent aneurysm – and images compatible with an arteriovenous fistula in this topography (Figure 3). The fistula path was catheterized via the right venous access with a 5Fr JR diagnostic catheter and 0.035 hydrophilic guide wire, which was later substituted for a 260 cm extra stiff 0.035 Amplatz guide wire. A 45cm 12Fr Flexor Check-Flo sheath (Cook) was positioned through the fistula orifice, via the right venous access. At this point a 21mm Figulla flex II vascular occluder (Occlutech) with two concentric discs was deployed, which successfully occluded the fistular communication between the aorta and the inferior vena cava (Figure 4). The occluder size was chosen on the basis of the size of the fistula orifice, which had been measured on initial angiotomography and angiography, and was oversized in order to guarantee good apposition against the degenerated aorta wall, to prevent migration.

**Figure 3:** Fistular path catheterized with 5Fr JR catheter via right venous access.

**Figure 4:** Figulla II occluder in place after release.

The infrarenal abdominal aortic aneurysm was then repaired using a 36×20×166 mm Endurant endograft main body (Medtronic), deployed via the left arterial access, 16×16×124 mm and 16×24×82 mm contralateral extensions, and a 16×20×93 mm ipsilateral extension. Final angiography showed that the aneurysm had been successfully repaired, the renal arteries were patent and there were no leaks, even when simultaneous injections via the arterial and venous accesses were applied (Figure 5).

**Figure 5:** Final angiography with no evidence of leakage.

The patient recovered well during the postoperative period and was discharged on the fifth day, in good clinical condition and with the lower limb edema in regression. A control angiotomography at 30 days showed the endograft patent and no signs of leakage. It was also possible to observe that the inferior vena cava was patent and the occluder was correctly positioned and with no evidence of secondary thrombosis (Figure 6). It has been 1 year since treatment and unfortunately the patient refuses to attend any type of clinical follow-up or submit to imaging exams. Via telephone he states that he has no new complaints or related symptoms.

**Figure 6:** Angiotomography with 3D reconstruction at 30-day follow-up.

## DISCUSSION

Aortocaval fistula is a rare complication of infrarenal abdominal aortic aneurysms, which can occur in up to 4% of ruptured aneurysm cases.^1^ The first case report was published by Syme in 1831 and Cooley described successful surgical treatment in 1955.^2^

It is believed that increased tension against the aneurysm wall causes an inflammatory reaction and adhesion to the adjacent vein – generally the inferior vena cava – resulting in erosion of the walls and formation of the fistula.^3^ The classic presentation consists of an abdominal pulsating mass associated with thrill and “machinery murmur”, right heart failure and signs of venous hypertension. On rare occasions paradoxical pulmonary embolism (PPE) may be caused by thrombi from the aneurysm entering venous circulation.^4^ Other signs and symptoms include jugular stasis, dyspnea, pleural effusion, hepatomegaly, ascites, and hematuria.^5^

In view of their severity, aortocaval fistulae should be treated as soon as they are diagnosed. Conventional surgical treatment is associated with perioperative mortality ranging from 16 to 66%.^6^ This is because these are usually patients of advanced age with multiple comorbidities and cases are complicated by the systemic hemodynamic changes caused by a high throughput fistula.^7^ Detailed pre-anesthetic assessment of cardiopulmonary function and carefully controlled administration of fluids and control of blood pressure are essential to increase the likelihood of success and reduce complications, particularly when the fistula is occluded, when acute cardiac decompensation may occur.^8^

Endovascular techniques are attractive alternatives to conventional surgical treatment. A review of the literature published by Antoniou et al. in 2009 reported a 96% technical success rate for endovascular treatment, with no reports of perioperative mortality within 30 days.^9^ However, there are certain theoretical concerns related to endovascular treatment. First, manipulation of the aneurysm lumen could provoke displacement of thrombi and result in a PPE. Additionally, treatment of the aneurysm without occlusion of the fistula could predispose to leakage, because of persistence of the fistula canal.^10^

A PPE is a rare event, but one that is associated with high morbidity and mortality.^11^ Since it is difficult to diagnose and can be confused with the patient’s symptoms of heart failure, it may be underestimated.^12^ Some reports describe temporary placement of a vena cava filter to prevent paradoxical embolism during manipulation of the lumen of the aneurysm to deploy the endograft.^13^^,^ 14 However, this practice is uncommon in the literature. Other reports only describe conventional treatment of the aneurysm with an endograft, without use of filters, achieving successful occlusion of the aortocaval fistula without reporting paradoxical embolism.^15^^-^ 17 In view of the size of the aneurysm exerting pressure on the wall of the vena cava (which could make placement and removal of the temporary filter difficult) and since the occluder was available, we decided not to employ a filter. When occluding the fistula canal prior to introduction of the endograft, we therefore manipulated the lumen of the aneurysm as little as possible to avoid displacement of thrombi, and consequently PPE.

A type II endoleak – which is fed by retrograde flow from branches of the aneurysm, typically a lumbar artery or inferior mesenteric artery – is the most frequently observed complication of endovascular treatment of aortocaval fistulae, seen in up to 22% of cases.^9^ However, reports in the literature show that this event is normally self-limiting.^18^ This type of leakage appears to be subject to different dynamics, by which the low pressure of the venous system provides an exit route for retrograde flow from the aortic branches, reduces tension at the aorta wall and facilitates spontaneous resolution.^19^ However, some authors suggest that, even after release of the endograft, blood flow into the aneurysm sac may be exacerbated through the fistula, which would require a second procedure to repair. To address this, ElKassaby et al. and Silveira et al. proposed concurrent treatment of the aneurysm and the aortocaval fistula, using an endograft from the arterial and venous sides, which proved feasible and may be more effective than endovascular treatment of the aorta alone.^20^^,^ 21 In view of the size of the aneurysm and the fistula, we considered that there was a high risk of endoleak and so we decided to treat both during the same operation. If the fistula had not been occluded and a leak had occurred during follow-up, a different strategy would have been needed to treat it, probably involving use of further high-value materials and the risk to the patient that an additional invasive procedure would involve. Since the materials needed for treatment in a single operation were available, we judged this to be the safest option.

Although this application was off-label, the occluder was a good fit to the arterial and venous walls, fulfilling its role without causing major technical difficulties during placement and release, since the fistular path had been catheterized in advance. Vascular occluders have been used previously in patients with a narrow iliofemoral axis given percutaneous aortic valve implants, in whom creation of a fistular path between the vena cava and the aorta is an access option for larger diameter devices.^22^^,^ 23 Godart et al.^24^ and LaBarbera et al.^25^ have used Amplatzer occluders to treat aortocaval fistulae. However, in their reports these authors employed the occluder device as a remedial procedure in patients who had previously been treated with endografts or conventional surgery to repair abdominal aneurysms, but had exhibited persistent flow through the fistular orifice in follow-up.

Use of the vascular occluder in combination with a bifurcated endograft to treat this case of infrarenal abdominal aortic aneurysm with an aortocaval fistula was successful and immediate results were satisfactory. Further studies are needed to assess routine use of vascular occluders for treatment of aortocaval fistulae, including long-term follow-up. As endovascular materials continue to evolve, new occluders or endoprostheses exclusively for venous applications may become the first choice for treatment of aortocaval fistulae.
